# Enhancing Emergency Care: Capacity Building in Basic Life Support (BLS) for Accident and Emergency Staff at a Ghanaian Emergency Department

**DOI:** 10.1155/emmi/6860643

**Published:** 2025-04-08

**Authors:** Nana Serwaa Agyeman Quao, Janet Naki Opare, Abena Antwiaa Adom-Asomaning, Eugene Adomako, Yaa Darkowaa Appiah, Malwine Abena Obuobisah

**Affiliations:** Accident and Emergency Centre, Korle Bu Teaching Hospital, Accra, Ghana

**Keywords:** basic life support, cardiac arrest, cardiopulmonary resuscitation, knowledge, training

## Abstract

**Background:** Cardiac arrest, a sudden cessation of cardiac function, necessitates immediate life-saving measures such as cardiopulmonary resuscitation (CPR) to prevent death. Despite poor survival rates and neurological outcomes associated with cardiac arrest, early resuscitation can improve survival. In low- to middle-income countries like Ghana, there are limited data on CPR practices and outcomes, necessitating targeted training programs.

**Objective:** To assess the impact of a basic life support (BLS) training program on knowledge acquisition and training among staff in the Accident and Emergency Department of Korle Bu Teaching Hospital.

**Methods:** A comprehensive BLS training program was conducted over four (4) days and involved 128 staff. Following the American Heart Association (AHA) BLS guidelines, the training sessions included lectures, practical sessions with adult and child CPR mannequins, and pre- and post-training online assessments and evaluations.

**Results:** Most participants were between 31 and 40 years old with the majority being nurses. The mean score for the post-test (13.95) was higher than that for the pretest (12.40). A total of 99% of the respondents agreed that their learning objective for the course was met and exceeded. A total of 74.71% responded that their knowledge of BLS had improved, with nearly 99% agreeing that the instructions for the practical sessions were clear and easy to follow. About 60% of the respondents rated the quality of delivery of the lectures excellent, with 85% satisfied with the time allocated to the training sessions. All participants indicated they would recommend the BLS training course to their colleagues.

**Conclusion:** This study strongly advocates for the integration of ongoing BLS training programs to maintain high standards of emergency medical care, particularly in resource-limited settings as regular BLS training can improve emergency care and patient outcomes.

## 1. Introduction

Cardiac arrest is the sudden cessation of effective cardiac function in a person [1]. This manifests as no pulse and no breathing in the affected patient. If the person does not receive immediate lifesaving support measures such as cardiopulmonary resuscitation (CPR), that person dies. CPR consists of the use of chest compressions and artificial ventilation to maintain circulatory flow and oxygenation during cardiac arrest [2]. The purpose of CPR is to restore partial flow of oxygenated blood to the heart and brain by delaying permanent tissue death to allow for successful resuscitation [3]. Cardiac arrest has poor survival rates and neurological outcomes; nonetheless, early resuscitation, including early defibrillation and effective post-cardiac arrest care, can lead to improved survival and neurological outcomes [2].

Cardiac arrest carries a high mortality rate. It is the third leading cause of death for out-of-hospital cardiac arrest in the US [1]. Out-of-Hospital Cardiac Arrest (OHCA) carries a survival rate of less than 8% [4]. According to the American Heart Association (AHA) 2020 guidelines, less than 40% of adults receive lay rescuer-initiated CPR, and fewer than 12% have an Automated External Defibrillator (AED) applied before Emergency Medical Services (EMS) arrival [5]. Statistics on bystander CPR are limited for low- and middle-income countries, such as African nations, and even more so for Ghana [4]. In these regions, morbidity and mortality from cardiovascular diseases are increasing and improving the performance of bystander or OHCA CPR could enhance survival rates from cardiovascular events [4].

The AHA has developed basic life support (BLS) training programs for health professionals and the public. BLS training involves teaching high-quality CPR and the use of an AED. In high-income countries, health staff and the public receive regular CPR training. Hospital emergency departments are manned by health professionals who are trained in BLS and are regularly certified.

In Africa, this resuscitation training is not widespread or available to everyone. With the introduction of emergency medicine in some African countries, including Ghana, some health facilities have organized this training for their staff, including doctors and nurses working in their emergency rooms. In Ghana, according to the policy guidelines for hospital Accident and Emergency services, all doctors and nurses in the emergency units must be trained in BLS, and Advanced Cardiac Life support (ACLS) and all other staff must be trained in BLS [6]. Few studies have been conducted to look at the impact of BLS training for health staff in Africa. For example, in the assessment of nurses' knowledge and skills in CPR in a Ugandan hospital, there was significant improvement in knowledge and skills after training [7].

During the 2024 World Emergency Day celebrations at the Accident and Emergency Center of Korle Bu Teaching Hospital, the department organized BLS refresher course for all staff, including doctors, nurses, and administrative personnel, to enhance their knowledge and skills.

## 2. Methods

The Accident and Emergency Department of the Korle Bu Teaching Hospital, as part of its 2024 World Emergency Medicine Week celebrations held from Monday, 27^th^ May to Friday, 31^st^ May 2024, organized a BLS training session for its staff. The purpose of the training was to enhance the resuscitation skills, mainly CPR of Emergency Department (ED) staff. The Accident and Emergency Department is one of the 21 departments in the hospital. It serves as the receiving point for trauma, medical, and surgical emergencies. The total staff strength is 326, comprising doctors, nurses, pharmacists, and administrative staff.

The training sessions were held at the Accident and Emergency Conference Room from Tuesday, May 28, 2024, to Friday, May 31, 2024. The BLS training was conducted according to the AHA BLS training guidelines. Staff were at liberty to choose which training day was convenient for them. Two sessions were held daily, with an average of 15–30 participants per session. Emergency Physicians from the Accident and Emergency Department conducted the BLS training.

Participants were required to take pre- and post-test questions via Google Forms. The 15 pretest single-choice answer questions were taken before the start of the training session. The questions were administered online using a unique QR code posted in the classroom. After the training session, participants were required to scan another QR code to access the 15 post-test single-choice answer questions. The content of the training focused on the importance of BLS, a chain of survival concepts, the performance of high-quality CPR, the use of AED/manual defibrillator, the performance of proper airway management in In-Hospital Cardiac Arrest (IHCA), the importance of team dynamics and debriefing, and relief of choking. Training videos were incorporated into the training sessions, and staff had the opportunity to practice good chest compressions with proper Bag-Mask-Ventilation (BVM) ventilation and the use of AED on available CPR mannequins.

Participants were required to fill out an online evaluation form using a different QR code after the training session to assess the performance of the BLS training. The evaluation sought to assess training expectations and objectives, knowledge improvement, relevance and usefulness of course, faculty assessment, practice session assessment, and suggestions and recommendations for future training.

Ethical approval was not required, as this study was conducted retrospectively following a BLS training session in the ED. The study evaluated data collected during training, which did not include interventions or research activities requiring direct ethical oversight.

The data was exported into Microsoft Excel and cleaned. The data were analyzed descriptively and presented in frequency tables and graphs. The 15 pre- and post-test questions each scored one point, with a maximum score of 15. A paired *t*-test was conducted to analyze the pre- and post-training mean scores, with significance at *p* < 0.05.

## 3. Results

**Figure 1:**
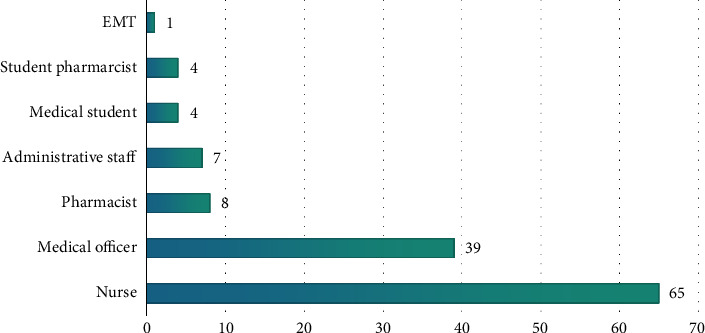
Cadres of staff.

**Figure 2:**
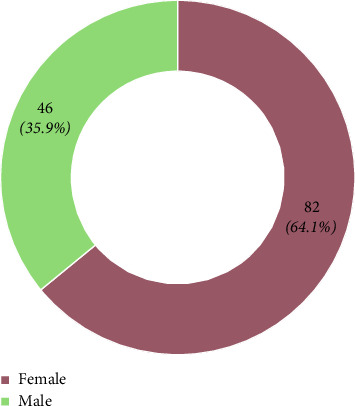
Gender of the participants.

A total of 221 staff participated in the BLS training, representing 67.8% of A&E staff, but due to technical challenges, only 128 were able to access the online tests.  Figure 1 shows the distribution of staff cadres. The majority of the participants were nurses, with 64.1% being females (see Figure 2). Most participants were between 31 and 40 years old (see Table 1).

The mean score for the post-test (13.95) was higher than that for the pretest (12.40), as seen in Table 2. The results also show that knowledge of BLS increased by 10.3%. The difference in knowledge tests between pre and post-training on BLS was statistically significant (*p* < 0.05), approximately 0.001. Almost all respondents agreed that their overall expectations were met and exceeded in content and delivery. Nearly 99% agreed that their learning objectives for the course were met and exceeded. A total of 74.71% responded that their knowledge of BLS had improved, with almost 99% agreeing that the instructions for the practical sessions were clear and easy to follow. When asked to rate the relevance and usefulness of the course content of BLS, 64.37% rated it as excellent and 29.89% as outstanding.  Table 3 shows the summary of the evaluation of the training.

The majority of respondents (95.40%) thought the level of complexity of the course was at the right level. About 60% of the respondents rated the quality of delivery of the lectures as excellent, with 85% satisfied with the time allotted for the training sessions. The level of interaction between facilitators and participants was rated positively at 60.92%. Around two-thirds of participants indicated that the training would enhance their ability to manage cases in the wards. All participants (100%) indicated they would recommend the BLS training course to their colleagues, as shown in Table 4.

## 4. Discussion

The outcomes of the BLS refresher course held at the Accident and Emergency Center of Korle Bu Teaching Hospital during the 2024 World Emergency Day celebrations underscore the necessity of regular training for healthcare providers. The significant increase in post-test scores compared to pretest scores indicates that such training effectively enhances the knowledge and skills of medical staff. The mean score improvement from 12.40 to 13.95 out of 15 reflects a 10.3% increase in BLS proficiency among participants. This outcome is similar to the results obtained in a study by Owojuyigbe et al., where the post-test results were significantly higher than the pretest results [1]. This reinforces the importance of regular training among healthcare providers to increase their knowledge and skills.

The study showed a substantial improvement in knowledge; however, this did not necessarily result in enhanced clinical effectiveness during actual patient care. Assessing the bedside impact of acquired knowledge is crucial. While the findings indicate increased knowledge, future research should examine whether this leads to enhanced CPR performance and better patient outcomes in emergency settings.

The high levels of satisfaction and perceived knowledge improvement reported by the participants in this study strongly support the effectiveness of the training. Nearly all participants (98.8%) acknowledged improved BLS knowledge, suggesting that the course content, duration, and delivery were well suited to their needs. Effective BLS training is critical for improving immediate patient outcomes and fostering a culture of preparedness and confidence in handling emergencies presenting to the emergency room. A study conducted in Uganda by Munezero et al. [7] found similar results. It affirmed that regular effective BLS training is necessary to maintain CPR competency and improve survival rates of patients experiencing cardiac arrest [7].

One key element emphasized during this training was the importance of adequate resources to facilitate effective learning. Training mannequins and AEDs are essential tools that provide hands-on practice opportunities, enhancing the realism and effectiveness of the training. The tactile experience of practicing on mannequins and using AEDs in simulated cardiac arrest scenarios helps to solidify theoretical knowledge and build confidence in performing life-saving procedures. The use of these tools significantly boosted the effectiveness of the training at Korle Bu Teaching Hospital. By allocating resources to these practical training tools, institutions can ensure that their healthcare personnel are not only knowledgeable but also proficient in the execution of essential BLS skills, ultimately leading to improved patient outcomes and a higher standard of emergency care.

In low-resource settings, making BLS training mandatory for all emergency unit staff is crucial. The constraints of limited medical resources, such as a lack of advanced life-saving equipment and longer response times for emergency services, make it essential that emergency unit staff are proficient in BLS. Mandatory training ensures that all personnel have the necessary skills to provide immediate and effective care, which can be lifesaving. Regular certification and recertification can help maintain high standards of emergency care and ensure that staff remain updated on the latest protocols and techniques [1].

In addition, BLS training should be integrated into medical school curricula. Early and repeated exposure to BLS techniques will ensure incoming healthcare professionals enter the workforce with a strong foundation in these critical skills [1]. This approach not only improves individual preparedness but also enhances the overall emergency response capabilities of healthcare institutions.

In addition to training healthcare professionals, educating the general public in BLS is equally important to improve bystander CPR. Widespread public education on BLS can significantly increase the chances of survival in out-of-hospital cardiac arrest cases [4]. An initial study conducted among the general public in Ghana showed that more than 90% of the nonhealthcare participants had never received CPR training; however, the majority were very willing to get trained [4]. Training members of the public in communities, schools, workplaces, and other public spaces in basic life-saving techniques will satisfy this need and ensure that more people can provide immediate assistance before professional medical help arrives. This community-level preparedness can bridge the gap in emergency response, especially in resource-limited settings.

## 5. Limitations of the Study

A significant limitation of the study is the disparity between the number of staff members who participated in the BLS training and those who completed the online tests and evaluations. While 221 staff members participated in the training, only 128 were able to access the online tests due to technical challenges. This reduction in the number of participants for the evaluation phase may have introduced bias, potentially leading to an overestimation of training effectiveness. The exclusion of nearly half of the participants from the post-training assessment limits the study's ability to fully capture the impact of the BLS training program across all staff members. Future studies should explore strategies to mitigate such data loss and assess its impact on the validity of training outcomes.

## 6. Conclusion

Consistent BLS training for healthcare personnel and providing adequate resources such as training mannequins and AEDs are vital for enhancing patient outcomes. Hands-on training resources are critical for skill acquisition and should be prioritized in all BLS training programs. Incorporating BLS training into medical education ensures that new graduates are equipped with critical life-saving skills from the start of their careers. The experience at Korle Bu Teaching Hospital serves as a model for other institutions seeking to strengthen their emergency care capabilities through continuous education and resource allocation.

## Figures and Tables

**Table 1:** Age distribution of the participants.

Age distribution	Number
21–30 years	38
31–40 years	82
41–50 years	6
51+ years	2
Total	128

**Table 2:** Summary of the paired *t*-test for the mean pre and post-test scores.

Pretest–post-test								
Paired differences			*t*-test	Significance level		Paired samples statistics		
Mean	Std. deviation	Std. error mean		One-sided *p*	Two-sided *p*	Mean		Std. deviation
1.554	1.758	0.175	−8.88	< 0.001	< 0.001	Pretest	12.40	1.727
						Post-test	13.95	1.203

**Table 3:** Summary of BLS Evaluation I.

**Overall expectations and objectives**	**Did not meet my expectations at all**	**Barely met my expectations**	**My expectations were met (%)**	**My expectations were exceeded (%)**	

Overall, to what extent did the BLS training meet your expectations by way of content and delivery?	0.00	0.00	59.77	40.23	
To what extent has your learning objective for the BLS training been met?	0.00	1.15%	71.26	27.59	

**Knowledge improvement and practical instructions**	**Strongly disagree**	**Disagree**	**Neutral**	**Agree (%)**	**Strongly agree (%)**

As a result of this training, my knowledge in basic life support has improved:	1.15%	0.00	0.00	24.14	74.71
The instructions for the practical sessions were clear and easy to follow:	0.00	0.00	1.15%	43.68	55.17

**Course content and delivery**	**Unsatisfactory**	**Satisfactory (%)**	**Good (%)**	**Excellent (%)**	**Outstanding (%)**

How would you rate the relevance and usefulness of the course content of the BLS training course?	0.00	2.30	3.45	64.37	29.89
How would you rate the quality of delivery of lectures for the BLS course?	0.00	1.15	5.75	59.77	33.33
How would you rate the level of interaction between faculty and participants in the BLS training course?	0.00	1.15	10.34	60.92	27.59
How would you rate your degree of satisfaction with the contribution that BLS training will make to your management of cases on the ward?	0.00	2.30	12.64	62.07	22.99

**Table 4:** Summary of BLS Evaluation II.

**Time allocation**	**Too little (%)**	**Adequate (%)**	**Too much (%)**

How would you rate the overall time period allotted to the lecture session of the BLS training?	9.20	85.06	5.75
How would you rate the overall time period allotted to the practical sessions of the BLS training?	19.54	78.16	2.30

**Course complexity**	**Too advanced**	**At the right level**	**Too basic**

How would you rate the level of complexity of the BLS course?	3.45%	95.40%	1.15%

**Recommendation**	**Yes**	**No**	**Maybe**

Would you recommend this course to your fellow colleague?	100%	0	0

## Data Availability

The data that support the findings of this study are available from the corresponding author upon reasonable request.
